# 

**Published:** 1892-08

**Authors:** 


### CONTENTS.

COMMUNICATIONS.

Syphilitic Disease of the Tongue........ 361
Micro-Organisms of the Mouth........ 368
Formation and Care of the Teeth........ 384
Immediate Root Filling........ 394
Pulp Protection by Cavity Lining........ 396
Dentists and Dentistry as Seen by Our Patients........ 402

PROCEEDINGS.

Vermont State Dental Society........ 405

COMMENCEMENTS.

German-American Dental College........ 407
University of Pennsylvania........ 408
Boston Dental College........ 409
Harvard University........ 409
University of Michigan........ 410
Howard University........ 410
University of Minnesota........ 411

EDITORIAL.

Washington State Dental Society........ 411
Sterilization of Metallic Instruments........ 412

